# Decellularization of xenografted tumors provides cell-specific *in vitro* 3D environment

**DOI:** 10.3389/fonc.2022.956940

**Published:** 2022-08-18

**Authors:** Gaia Iazzolino, Unai Mendibil, Blanca Arnaiz, Ane Ruiz-de-Angulo, Mikel Azkargorta, Kepa B. Uribe, Neda Khatami, Felix Elortza, Beatriz Olalde, Vanessa Gomez-Vallejo, Jordi Llop, Ander Abarrategi

**Affiliations:** ^1^ Center for Cooperative Research in Biomaterials (CIC biomaGUNE), Basque Research and Technology Alliance (BRTA), Donostia-San Sebastian, Spain; ^2^ TECNALIA, Basque Research and Technology Alliance (BRTA), Donostia-San Sebastian, Spain; ^3^ Center for Cooperative Research in Biosciences (CIC bioGUNE), Basque Research and Technology Alliance (BRTA), Derio, Spain; ^4^ Ikerbasque, Basque Foundation for Science, Bilbao, Spain

**Keywords:** tumor xenograft, decellularization, *in vitro*, 3D model, breast cancer

## Abstract

*In vitro* cell culture studies are common in the cancer research field, and reliable biomimetic 3D models are needed to ensure physiological relevance. In this manuscript, we hypothesized that decellularized xenograft tumors can serve as an optimal 3D substrate to generate a top-down approach for *in vitro* tumor modeling. Multiple tumor cell lines were xenografted and the formed solid tumors were recovered for their decellularization by several techniques and further characterization by histology and proteomics techniques. Selected decellularized tumor xenograft samples were seeded with the HCC1806 human triple-negative breast cancer (TNBC) basal-like subtype cell line, and cell behavior was compared among them and with other control 2D and 3D cell culture methods. A soft treatment using Freeze-EDTA-DNAse allows proper decellularization of xenografted tumor samples. Interestingly, proteomic data show that samples decellularized from TNBC basal-like subtype xenograft models had different extracellular matrix (ECM) compositions compared to the rest of the xenograft tumors tested. The *in vitro* recellularization of decellularized ECM (dECM) yields tumor-type–specific cell behavior in the TNBC context. Data show that dECM derived from xenograft tumors is a feasible substrate for reseeding purposes, thereby promoting tumor-type–specific cell behavior. These data serve as a proof-of-concept for further potential generation of patient-specific *in vitro* research models.

## Introduction

Understanding the biology of tumor initiation and progression processes has a huge impact on the successful development of novel and effective cancer treatments (1). Despite its high cost and ethical concerns, *in vivo* human tumor cell xenografting in mouse orthotopic and ectopic locations remains the most reliable experimental model to mimic tumor microenvironment and to study, among others, tumor formation and progression processes (2). Moreover, *in vitro* monolayer culture of cancer cells in a two-dimensional (2D) environment is a highly popular and straightforward method to perform cancer-related preclinical research (3). However, 2D cell cultures are considered too simplistic to accurately test solid tumor-derived cell behavior, as they do not resemble the tumor’s three-dimensional (3D) architecture and the lack of mechanical/biochemical signals coming from 3D cell–cell and cell–matrix interactions (4, 5).

In comparison to 2D cell cultures, 3D cell culture models are more accurate tumor-mimicking approaches (6) because they present enhanced cell–matrix and cell–cell interactions while modulating differently those signaling pathways related to cell migration, morphology, proliferation, and viability (7–10). Among *in vitro* cancer 3D models, those promoting extracellular matrix (ECM) and cell interaction are considered the most relevant. For example, hydrogels formed with the ECM extracted for cell-cultured tumor cells (e.g., Matrigel) are widely exploited in cancer research as matrices for tumor cell seeding and study (3, 11). These hydrogels provide chemical and physical characteristics potentially beneficial to mimicking tumor 3D environment, as they have ECM structural proteins (e.g., collagens) to which cells can attach, along with growth factors, hormones, and other essential molecules (12). Despite having noteworthy properties, commercially available cell culture extracted ECM and their hydrogels possess various significant drawbacks. Most notably, by using this commercial ECM as a 3D culture environment, the target cells are exposed to an ECM composition different from the one of the specific tumor to be studied. Moreover, the cells at hydrogels grow as spheroids, and therefore, they lose their original morphology, mobility, and cell–cell and cell–matrix interactions. Furthermore, from a technical point of view, the postprocessing steps required after decellularization may induce a loss in the processed material and that is not assumable when handling small decellularized samples.

Aiming to define more reliable and tumor-specific 3D cancer models, bioengineered approaches have recently been explored. For example, hydrogels have been structured using 3D bioprinting technology to generate matrices or microfluidic cancer-on-a-chip models. On the other hand, spontaneous spheroids formed by target cells have been considered a potential valid *in vitro* tumor-mimicking model, while tumor organoids formed by the multiple cell types present in the targeted tumor are the ultimate promise to mimic tumor complexity in *in vitro* structures (13–21).

Decellularization is a propitious approach in which cells are removed from tissues or organs, thereby isolating the ECM structure to be recovered (22). This technique provides an advantageous approach to producing more effective tissue models in which ECM and tissue architecture are preserved (23). The advantage of using biologically derived matrices for *in vitro* 3D studies is that some part of the major proteins and factors already exist in the decellularized scaffold, which makes them useful in a wide range of applications, from physiological regenerative medicine to pathological (cancer) research studies (24–30).

Decellularized ECM (dECM) can be obtained from both normal and tumor tissues following several types of decellularization protocols, including physical, chemical, or enzymatic methods reported in the literature. The different decellularization methods alter the ECM components differently, and therefore, it is important to define the tissue-specific decellularization method in each context (31). dECM can then be used as a 3D scaffold or it can be processed for hydrogel formation (32, 33). Matrices from normal tissue can be used to recognize novel genes inducing cancer or the interactions between cancer cells and healthy ECM. On the other hand, decellularized tumor tissue is more appropriate for studying the influence of pathological ECM on controlling cancer cell function at primary or metastatic sites and the role of ECM as a modulator of cell behavior (34–37).

In this manuscript, we hypothesized that decellularized xenograft tumors can serve as an optimal 3D substrate to generate a top-down approach for *in vitro* tumor modeling. Aiming to prove that, we xenografted multiple tumor cell lines and the formed solid tumors were recovered for their decellularization by several techniques. Data indicate a soft decellularization protocol based on tumor freezing followed by EDTA treatment allows the recovery of dECM with tumor-type–specific protein components. When used as a substrate for human triple-negative breast cancer cell seeding, and compared to other 3D cell culture models, a characteristic cell behavior was observed in terms of cell morphology, proliferation, and mobility.

## Materials and methods

### Cell cultures

Cell lines were numbered from 1 to 13 for easy trackability of samples during the study (see **Table 1**). MDA-MB-231 (RRID : CVCL_0062), MIA PaCa-2 (RRID : CVCL_0428), PANC-1 (RRID : CVCL_0480), U87 (RRID : CVCL_0022), HepG2 (RRID : CVCL_0027), and Du145 (RRID : CVCL_0105) cell lines were cultured in DMEM (11995065; Gibco, UK); 4T1 (RRID : CVCL_JG34), HCC1806 (RRID : CVCL_1258), and AR42J (RRID : CVCL_0143) cell lines were cultured in RPMI-1640 (11875101; Gibco, UK); Capan1 (RRID : CVCL_0237) and Pc3 (RRID : CVCL_0035) cell lines were cultured in F12K (21127022, Gibco, UK); and GH4 (RRID : CVCL_WX20) cell line was cultured in Ham’s F10 (11550043, Thermo Fisher). All media were supplemented before being used with 10% fetal bovine serum (10500-064, Gibco, UK), 2 mM l-glutamine (25030024, Gibco, UK), and 50 U/ml penicillin and 50 µg/ml streptomycin (15070063, Gibco, UK). As an exception, AR42J cells required 20% fetal bovine serum as a supplement.

**Table 1:** Tumor cell lines, origin, and provided number.

Cell line number	Cell line name	Tumor tissue	Organism	Number (*n*) of implants	Time for engraftment (days)	Volume at harvest (mm^3^)
**1**	MDA-MB-231	Breast	Human	6	45	89 ± 37
**2**	4T1	Breast	Mouse	6	7	463 ± 205
**3**	HCC 1806	Breast	Human	6	15 ± 6	321 ± 82
**4**	AR42J	Pancreas	Rat	7	28 ± 1	1,065 ± 390
**5**	Capan 1	Pancreas	Human	6	60 ± 7	223 ± 122
**6**	MIA PaCa-2	Pancreas	Human	6	43 ± 15	310 ± 258
**7**	PANC-1	Pancreas	Human	7	45	138 ± 42
**8**	Du145	Prostate	Human	7	33 ± 1	133 ± 25
**9**	PC-3	Prostate	Human	7	45	240 ± 66
**10**	U-87	Brain	Human	7	42 ± 3	1,140 ± 379
**11**	HepG2	Liver	Human	7	45	302 ± 196
**12**	PC-12	Adrenal gland	Rat	6	35 ± 5	504 ± 210
**13**	GH4	Pituitary	Rat	6	21 ± 5	262 ± 39

### Xenografted tumor generation

Animals were cared for and handled following internal guidelines and in compliance with the European Guidelines for Accommodation and Care of Animals. The study was conducted in accordance with the Declaration of Helsinki and approved by the Ethics Committee of CICbiomaGUNE and local authorities (protocol code PRO-AE-SS-166 and date of approval 01/06/2019).

Cells were resuspended in Matrigel HC (354248, Corning, USA) diluted 1:4 in cold Dulbecco’s phosphate-buffered saline (DPBS, 14040-091, Gibco, UK) and injected in the back (100 µl/animal) of 6–8-week-old athymic nude mice (Crl : NU(NCr)-Foxn1nu, Charles River). Animals were monitored for tumor growth using an electronic digital caliper 779A series (Starrett) (see **Table 1**). At the endpoint, animals were processed for perfusion with heparinized physiological serum. The tumors were extracted, deposited in a sterile container, and stored at −80C.

### Decellularization protocol

The tumors were thawed and sectioned into 30 mg pieces for further decellularization. Six custom protocols were used in the initial studies (see **Table 2**).

**Table 2:** Treatment type and concentrations used in each one of the decellularization protocols.

Protocol	DNase (U/ml)	Freezing (cycles)	Trypsin (% w/v)	Triton X-100 (% w/v)	EDTA (% w/v)
**a**	–	15 cycle	–	–	–
**b**	50 U/ml	–	–	–	–
**c**	50 U/ml	–	0.25%	–	0.25%
**d**	50 U/ml	–	–	–	2%
**e**	50 U/ml	15 cycle	–	–	2%
**f**	50 U/ml	–	–	1%	–

Protocol “a” corresponds to 15 freezing cycles. Protocol “b” corresponds to three washes of DNase (Sigma-Aldrich) at 37°C in agitation. Protocol “c” uses Trypsin-EDTA (25050-014, Sigma-Aldrich, USA) over 30 min at 37°C in agitation, followed by the DNase (7469, StemCell, CA) treatment mentioned in protocol b. Protocol “d” uses EDTA (E9884, Sigma-Aldrich, USA) for 30 min at room temperature (RT) in agitation, followed by the DNase treatment mentioned in protocol b. Protocol “e” uses the freezing cycles of protocol a, followed by treatment mentioned in protocol d. Protocol “f” uses Triton X-100 (T9284, Sigma-Aldrich, USA) for 30 min at RT in agitation, followed by the DNase treatment mentioned in protocol b. In all protocols, each step was followed by three DPBS washes in agitation for 20 min at RT.

### DNA extraction and quantification

To verify the decellularization efficiency, the DNA was extracted and quantified. The extraction was performed using the EchoLUTION Tissue DNA Micro Kit (010-002-010, BioEcholife), and for the DNA quantification, a NanoDrop (Jasco V-730) spectrophotometer was used. Data were obtained as nanograms of DNA per milligram of the wet original tissue. According to the ASTM International standards, 50 ng of DNA/mg of tissue was established as the acceptable DNA threshold to consider the tumor as decellularized (38).

### Histology of the decellularized tissues

Samples were embedded in Tissue Tek OCT (4583, Sakura Finetek, Japan), stored at −20°C, and sliced in the cryostat (CM1860, Leica). To check the nuclei distribution changes and the overall ECM structure, the slides were fixed for 1 h with neutral buffered 10% formalin solution (HT501128, Sigma-Aldrich, USA) and stained with hematoxylin (5 min) and eosin (2 min) (05-M06015 and 05-M10003, Bio-Optica, It).

Collagens and noncollagenous proteins of dECM were visualized by staining the samples with the Sirius Red/Fast Green Staining kit (9046, Chondrex, USA). This is a differential staining with two dyes, as Sirius Red binds to all types of collagen, whereas Fast Green stains noncollagenous proteins. All histology samples were visualized on a routine optical microscope equipped with a camera.

### Scanning electron microscopy

The decellularized samples were frozen at −20°C for 24 h and subsequently lyophilized for 24 h. A sputter coater (Alto 1000, Galan) was used to coat the sample’s surface with gold/palladium before their visualization in JSM-6490LV (JEOL) scanning electron microscope (SEM) equipment.

### Proteomics and mass spectrometry

Proteins were extracted from decellularized samples using a mixture of 7 M urea, 2 M thiourea, 4% CHAPS, and 5 mM DTT and then digested following the filter-aided FASP protocol described by Wisniewski et al. (39) with minor modifications. Briefly, trypsin was added to a trypsin:protein ratio of 1:20, and the mixture was incubated overnight at 37°C, dried out in a RVC2 25 speedvac concentrator (Christ), and resuspended in 0.1% FA. Peptides were desalted and resuspended in 0.1% FA using C18 stage tips (Millipore).

Samples were analyzed on a timsTOF Pro with PASEF (Bruker Daltonics) coupled online to an Evosep ONE liquid chromatograph (Evosep). A total of 200 ng was directly loaded onto the Evosep ONE and resolved using the 30 sample-per-day protocol.

Protein identification and quantification were carried out using MaxQuant software (40) using default settings except for the match between runs (match time window of 5 min, alignment tie window of 20 min) and an LFQ min. ratio count of 1. Searches were carried out against a database consisting of human and mouse protein entries (Uniprot/Swissprot), with precursor and fragment tolerances of 20 ppm and 0.05 Da. Only proteins identified with at least two peptides at FDR<1% were considered for further analysis. LFQ intensities were used for further analyses and loaded onto the Perseus platform (41).

Data were loaded onto the ClustVis web tool for the visualization and clustering of multivariate data using principal component analysis (PCA) (42). Data were loaded to MetaboAnalyst to be normalized and statistically analyzed. Partial least squares discriminant analysis (PLS-DA) was used to get the variable importance in projection (VIP) and differentiate the groups. For the Gene Ontology (GO) analysis ShinyGO web tool was used. All the results were represented using Graphpad Prism software.

The original contributions presented in the study are publicly available. The mass spectrometry proteomics data have been deposited to the ProteomeXchange Consortium. Data can be found here: [PXD034597].

### *In vitro* cell culture

HCC1806 (RRID : CVCL_1258) cell line cultured in RPMI-1640 was used for materials seeding studies. Control collagen scaffolds (20483, collagen wound dressing Suprasorb^®^ C, Lohmann&Rauscher, Austria) and tumor-derived dECMs were firstly cut into smaller pieces of approximately 3–10 mm and sterilized. Briefly, frozen tumor small pieces were thawed and two washes of ethanol (diluted to 70%, Et 00020005P, Sharlau, Spain) for 15 min were performed, followed by cleaning and rehydration by three washes at sterile DPBS for 5 min. Furthermore, UV light irradiation was applied for 18 h inside a cabinet (Bio II Advance Plus, Telstar, Japan). The cells were deposited similarly to what was previously described for other similar materials (43–45). Each material piece was transferred to a 48-well plate well (3548, Costar, USA) and injected with 30 µl of a cell dilution, trying to seed evenly all around the sample, resulting in 70,000 HCC1806 cells seeded per sample. In parallel, cells in sterile DPBS were mixed in a ratio of 1:4 with Matrigel^®^ to achieve a final concentration of Matrigel of 4 mg/ml for hydrogel formation into a 96-well plate (92096, TPP, Switzerland). Cell-seeded samples were incubated at 37°C and 5% CO_2_ for 1 h in a Forma Steri-cycle CO_2_ incubator (Model 381 Thermo Scientific, USA), then covered with complete warm complete RPMI-1640 media and finally cultured at 37°C and 5% CO_2_ for 3 days.

### Cell morphology and immunostaining

Actin fibers and nuclei were stained as follows. Samples were washed with DPBS and fixed in 10% formalin solution, neutral buffered (HT501128, Sigma-Aldrich, USA) for 30 min, washed with DPBS, and incubated with DPBS containing ActinRed555 ReadyProbes reagent (1/10 dilution, R37112, Invitrogen, USA) for 30 min at 37°C. Following two washes with DPBS, the scaffolds and cells were imaged in DPBS containing 5 ng/ml 2-[4-(aminoiminomethyl)phenyl]-1H-Indole-6-carboximidamide hydrochloride (DAPI) (D9542, Sigma-Aldrich, USA).

For immunolabeling of the Ki67 nuclear marker, samples were permeabilized by 5 min incubation in DPBS supplemented with 0.1% Triton X-100 (T9284, Sigma-Aldrich, USA) and washed for 5 min in DPBS. Blocking of unspecific binding sites was achieved by 30 min incubation with 1% bovine serum albumin (BSA, A7906, Sigma-Aldrich, USA) in DPBS containing 0.1% Tween 20 (DPBST, P9416, Sigma-Aldrich, USA). The Ki67 nuclear marker was then labeled by incubation with 1% BSA in DPBST containing rabbit anti-human Ki67 primary recombinant monoclonal antibody (1:200 dilution, MA5-14520 Invitrogen, USA) at 4°C overnight in a humidified chamber. Samples were washed three times with DPBS and incubated for 1 h in the dark with a donkey anti-rabbit antibody polyclonal highly cross-absorbed with Alexa Fluor Plus 488 (dilution 1:500, A32790, Invitrogen, USA) in 1% BSA DPBST. Samples were then washed with DPBS, stained with DAPI-containing (5 ng/ml) DPBS, and imaged. All the incubation times described above were doubled in the assays, including the Matrigel^®^ matrix.

For cell imaging, the scaffolds with fluorescently labeled cells were kept covered with DPBS in a 35-mm-diameter ≠1.5 optical glass bottom dish (D35-20-1.5-N, Cellvis, CA). Single plane images were taken in a Laser Scanning Confocal Microscope (Zeiss LSM 880, Carl Zeiss AG, Germany) with the Plan-Apochromat ×10/0.45 employing the excitation/emission wavelengths of 561 nm/610–715 nm for Actin Red 555, 405 nm/420–475 nm for DAPI, and 488 nm/500–600 nm for Ki67 labeled with Alexa Fluor 488 (AF488). For imaging the tumor-derived dECM scaffolds, 458 nm/441–471 nm or 633 nm/620–650 nm were used in reflection mode, while transmitted light was used in the case of collagen-based scaffolds or Matrigel^®^ matrix samples. In total, 20 to 40 µm Z-stacks were imaged, and maximum intensity z-tack projections were obtained. For quantification purposes, ×10 images were used, and data were normalized to the nuclei number in each image.

For the quantification of Ki64^+^ nuclei and the mean cell area, the FIJI image analysis software was employed. FIJI is a distribution of the popular open-source software ImageJ (National Institutes of Health, USA) focused on biological-image analysis (46).

To calculate the Ki64^+^ nuclei, immunostaining for Ki67 in green (AF488) and counterstaining of nuclei in blue (DAPI) were employed for proliferative nuclei stain and general nuclei stain, respectively (green and blue channels). Regions of interest (ROI, objects) were automatically drawn for the nuclei, and a mask is generated. The mask was overlaid on the image with the Ki67^+^ image. The percentage of proliferating cells was calculated as a ratio of Ki67^+^ objects to nuclei counts (total object number).

To calculate the mean cell area, ActinRed555 and DAPI stains were employed for cell and nucleus delimitation, respectively. Once the cells were delimited (objects), the area of the objects was measured. Finally, the mean cell area was calculated by dividing the total area of the objects by the total amount of nuclei in each picture.

### Live imaging

Complete RPMI-1640 media of samples in a 35-mm-diameter ≠1.5 optical glass bottom dish was replaced by warm complete RPMI-1640 media containing 1.5 to 2.5 µg/ml Calcein-AM (56496, Sigma-Aldrich, USA) and incubated for 30 min at 37°C and 5% CO_2_. Samples were transferred to a Laser Scanning Confocal Microscope (Zeiss LSM 880, Carl Zeiss AG, Germany) equipped with a ×20/0.8 objective lens and a chamber to keep conditions of 95% humidity, 37°C, and 5% CO_2_ during Z-stacks image acquisition period using 488 nm/500–600 nm excitation/emission wavelengths. Z-stacks are presented as maximum intensity Z-projection images employing the Zen Blue software v2.3 (Carl Zeiss AG, Germany).

For live time-lapse imaging, images were acquired for 5 h every 15 min employing the Axio Observer Z1 microscope (Carl Zeiss AG, Germany) with the EC Plan-NEOFLUAR 5×/0.16 Ph1 M27, the LED illumination module at 470 nm, and the 38 HE eGFP shift free filter set. In addition, during the acquisition, samples were kept at 37°C and 5% CO_2_ inside the equipment in an Incubator XL S1 (Carl Zeiss AG, Germany). Live cell single plane images were also acquired in the LSM 880 confocal microscope with a heating insert P S1 (130-800 005, PeCon, Germany) for temperature and atmosphere control. For video processing, the Zen Blue software v2.3 (Carl Zeiss AG, Germany) was employed. The screen recorder software OBS Studio v37.2.4 (^©^ 2022 GitHub, Inc. CA) was used for additional video editing. To measure cell displacement, the videos were analyzed with the FIJI plugin TrackMate v2.1.0 (47) that semiautomatically segments fluorescent cells present in the first frame and generate tracks that follow each cell through the rest of the video frames giving a score equal to the number of frames in which a particular cell was found. The resulted list, including the tracking number, frame number, and the position (*x*/*y*) of each cell in the image, is next analyzed by the Chemotaxis and Migration Tool 2.0 software (ibidi GmbH, Germany) to plot a Cell Tracking graph for the displacement of cells in a centered coordinate grid and to calculate the accumulated distance of each cell after the 5 h that lasted for the recording (image scaling, 3.87 µm per pixel).

### Statistics

All data were plotted and statistically analyzed using the Graphpad Prism software (La Jolla, CA, USA). In all cases, data normality was verified using the Shapiro–Wilk test and a normal QQ plot was assessed. Data that passed the normality test (alpha = 0.05) were statistically analyzed using parametric tests (ordinary one-way ANOVA) and Tukey’s multiple comparisons tests. Data that did not pass the normality test (alpha = 0.05) (data in **Figure 6G**) were analyzed using the Kruskal–Wallis nonparametric test and Dunn’s multiple comparisons tests. Data are presented as mean ± SD, and each individual point is provided in the plots. Additionally, the *n* number of each assay is provided in the figure legends. P values of less than 0.05 were considered statistically significant (ns *p* > 0.05; ^*^ *p* < 0.05; ^**^ *p* < 0.01; ^***^ *p* < 0.001).

## Results

### A soft treatment based on Freeze-EDTA-DNAse allows tumor decellularization

**Figure 1:**
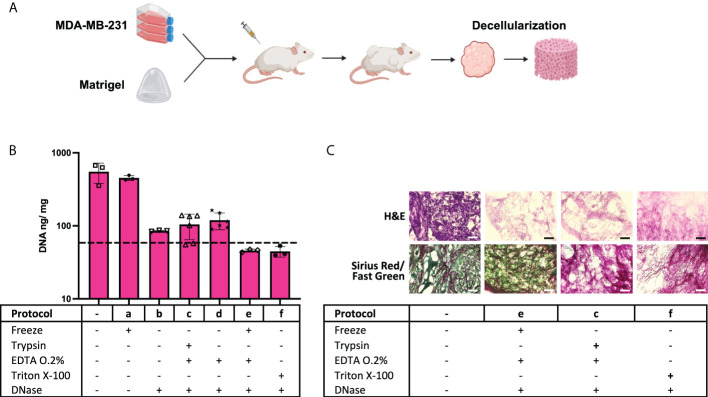
Enzyme and detergent-based protocols yield different decellularization outcomes. **(A)** Schematic of cell xenografting in mice: tumor formation, sample harvesting, and decellularization. **(B)** DNA quantification data on control samples and “a” to “f” protocol-treated samples (*n* = 3 to 6). The dotted line indicates the 50-ng DNA/mg tissue threshold used to define successful decellularization. **(C)** Histology assessment of samples. (Black and white scales represent 100 µm).

**Figure 2:**
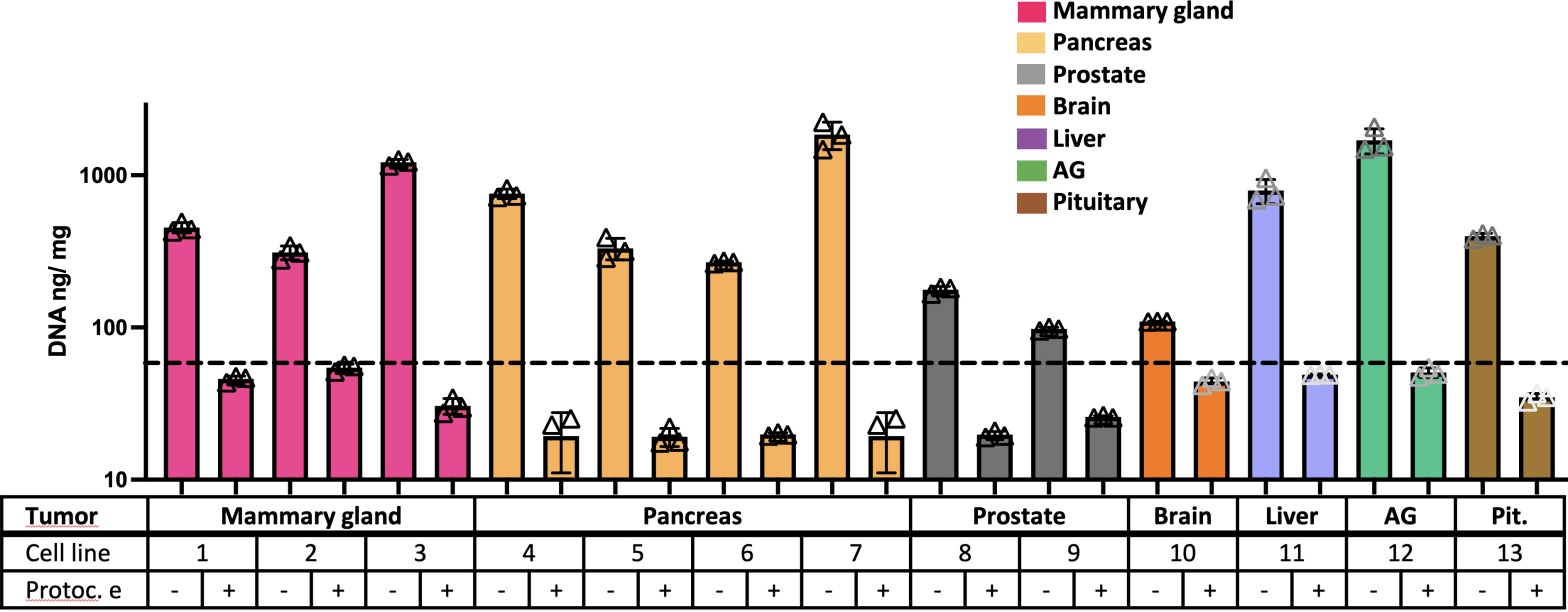
Freeze-EDTA-DNAse protocol decellularizes multiple tumor xenografts. DNA quantification data on control samples and “e” protocol-treated samples from multiple types of tumor xenografts (*n* = 3). The dotted line indicates the 50-ng DNA/mg tissue threshold used to define successful decellularization. Note: cell line number codes are provided in **Table 1**.

Tumor xenografts (**Figure 1A**) obtained from the MDA-MB-231 cell line, numbered as 1 in **Table 1**, were tested for decellularization with multiple custom-designed decellularization protocols (see **Table 2**). The DNA quantification results in **Figure 1B** (and **Supplementary Table S1**) show that protocols e and f successfully reduced the DNA content below the 50-ng DNA/mg tissue threshold. The histology examination in **Figure 1C**, H&E staining for nucleic acids and ECM, confirms the absence of hematoxylin-related purple nucleic acid staining in e and f protocol-treated decellularized samples but the presence of eosin-related ECM pink staining in all samples. In **Figure 1C**, Sirius Red/Fast Green dye combination staining is shown. It is used to distinguish collagen from its surrounding materials, as Sirius Red stains collagens in red, while Fast Green stains noncollagenous proteins in green. Samples treated with the protocol e (freezing cycles followed by EDTA) stain red and green similar to the control untreated samples. However, trypsin enzyme-treated and Triton X-100 detergent-treated samples showed an absence of the green color, which is typical of the ECM noncollagenous protein staining. Altogether, data in **Figure 1** indicate that the treatment of MDA-MB-231 cell line tumor xenografts with a protocol based on freezing cycles-EDTA-DNAse allowed for sample decellularization and proper preservation of tumor ECM in terms of collagens and proteins. Following these data, multiple types of tumor xenografts were processed using the decellularization protocol e. As it is shown in **Figure 2** (and **Supplementary Table S2**), in all cases, the treatment yields a successful xenograft tumor sample decellularization.

### Triple-negative breast cancer basal-like subtype xenograft tumors show a distinctive ECM protein composition after decellularization

**Figure 3:**
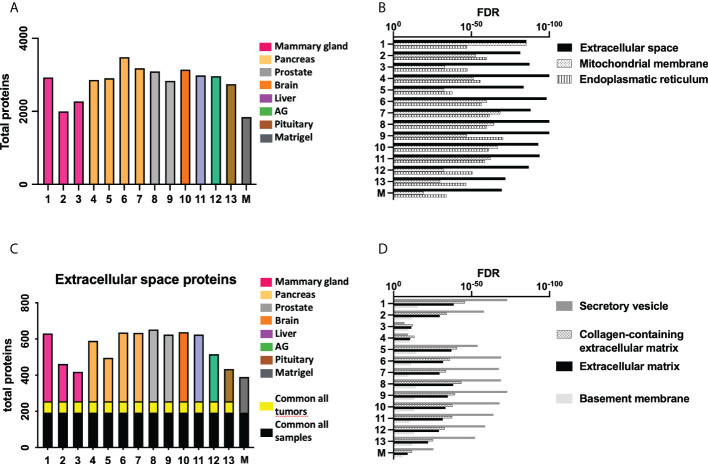
Proteomic study shows differences in ECM composition among samples. **(A)** Total amount of proteins detected in the proteomic study. **(B)** The most relevant Gene Ontology (GO) terms related to retrieved proteins in each sample. **(C)** The number of proteins from the GO term “Extracellular space” present in each sample, and the **(D)** GO terms related to these shortlisted proteins. Note: cell line number codes are provided in **Table 1**. The same tumor color codes are used in all panels of the figure.

A proteomic study was performed to define the composition of the decellularized ECM. Matrigel was included in the study as a control ECM. **Figure 3** (and **Supplementary Figure S1**) shows the number of total proteins detected in each kind of sample. A GO analysis revealed an enrichment of proteins related to “extracellular space” in all tested samples and also the existence of proteins from mitochondria and endoplasmic reticulum (ER), possibly related to cellular debris (**Figure 3B**).

A more detailed study of extracellular space proteins (**Figures 3C, D** **)** revealed a group of roughly 200 proteins commonly present in all samples, including control Matrigel. There was another group of proteins commonly present only in xenografted tumor samples, while all samples presented a high number of sample-specific extracellular space proteins. Further GO analysis (**Figure 3D**) revealed a specific enrichment on proteins related to secretory vesicles and extracellular matrix. The group of extracellular space proteins present in control Matrigel was compared to all other target tumor xenograft samples, aiming to identify ECM proteins down- or upregulated in xenografted tumors compared to the control Matrigel condition. In this sense, Matrigel showed a higher amount of Fibulin-1 and LAMA5 laminin subunit, two components previously related to the inhibition of tumor progression processes because they mediate different cell attachment, migration, and organization processes into tissues.

**Figure 4:**
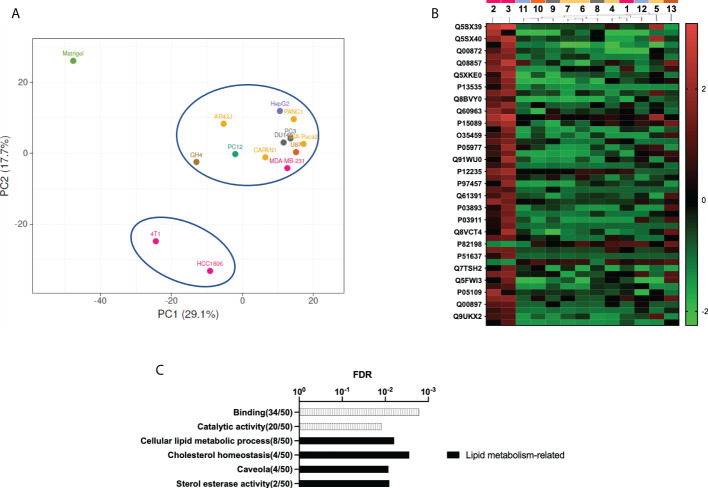
Proteomic study shows distinctive ECM on decellularized TNBC xenograft samples. **(A)** Principal component analysis (PCA). Matrigel control samples are plotted separately from all tumor xenograft samples. Tumor xenograft samples come out splinted into two groups, with the two TNBC xenograft-derived samples separated from the rest of the samples. **(B)** Heat map representation of the 50 more discriminant proteins obtained by partial least squares discriminant analysis (PLS-DA). **(C)** GO analysis of the 50 more discriminant proteins. Note: cell line number codes are provided in **Table 1**. The same tumor color codes are used in all panels of the figure.

The principal component analysis revealed again that all decellularized xenografted tumors had a protein composition different from the control Matrigel (**Figure 4A**). Moreover, this principal component analysis revealed that two triple-negative breast cancer xenograft-decellularized samples were different from all other samples. These samples correspond to triple**-**negative breast cancer (TNBC) basal-like subtype. A detailed study of discriminant differences between samples revealed the 50 more significant differentially present proteins in these two TNBC samples. The heat map representation of these proteins shows that most of them were overrepresented in these two TNBC samples, compared to all other decellularized xenografted tumor samples (**Figure 4B**). GO analysis of the 50 proteins indicates they were mainly proteins with catalytic activity and related to lipidic and cholesterol metabolism (**Figure 4C**). These differences may be related to the observed incomplete removal of cell mitochondrial and/or ER components, which would show up the differences in cell metabolism along the different tumor xenografts tested, a feature specifically relevant for TNBC basal-like subtype samples.

### HCC1806 TNBC cells show substrate-specific behavior when seeded on their tumor xenograft-derived dECM

**Figure 5:**
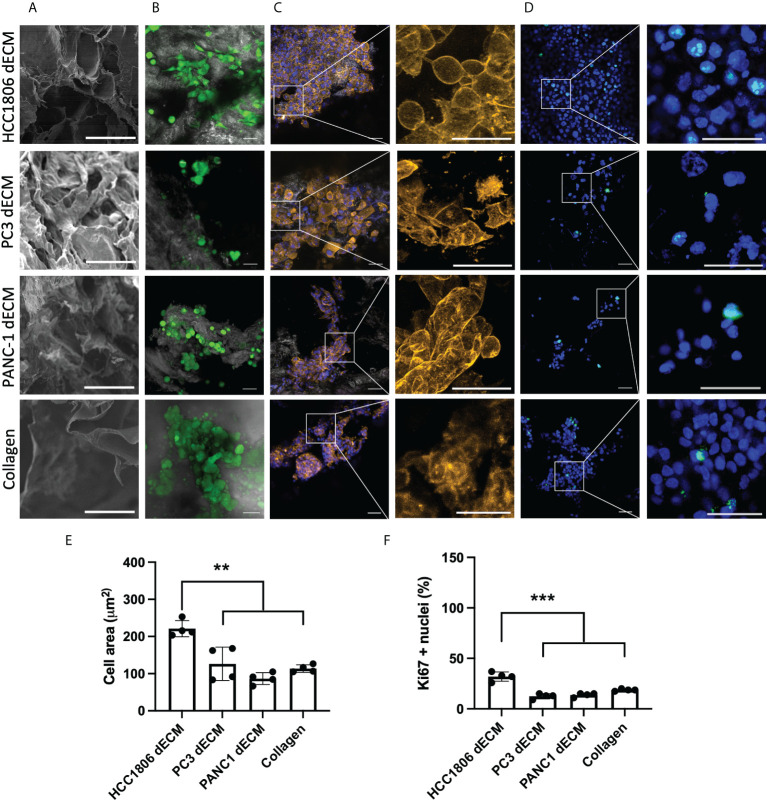
HC1806 cells show different cell behavior on HC1806 dECM compared to dECM from other tumor xenografts. **(A)** Detail of SEM imaging of samples showing surface structure (scale, 50 μm). **(B-D)** Images of cell-seeded samples. **(B)** Calcein-AM–positive cells are shown in green, while scaffold material is shown in grey (×20 (scale, 20 μm)). **(C)** Actin cytoskeleton immunostaining in orange; DAPI nuclear staining in blue (×10 (scale, 50 μm) and detailed images (scale, 20 μm)). **(D)** Ki67 immunostaining in green; DAPI nuclear staining in blue (×10 (scale, 20 μm) and detailed images (scale, 50 μm)). **(E)** Quantification of cell size (area) using actin cytoskeleton membrane distribution images. **(F)** Quantification of proliferative cells (Ki67-positive cells vs. total cells observed in each material). *N* = 3 in all cases and 2 areas measured per sample.

One of the potential applications of tumor dECM is as a substrate in cell culture studies to generate *in vitro* cancer models. As shown in **Figure 4C**, dECM from HCC1806 and 4T1-xenografted tumors show a differential composition in the proteomic PC analysis. At this point, we hypothesized that this specific protein composition may modulate cell behavior in *in vitro* studies. Therefore, we considered them a promising substrate with specific features to be tested in a cell culture context. Specifically, we wondered whether the cells that generated these tumors would behave differently on their specific tumor-derived dECM compared to other substrates. As 4T1 is a mouse cell line, we selected the HCC1806 human cell line to follow with *in vitro* cell culture studies and characterized the potential of the generated tumor dECM as *in vitro* model for cell culture studies. First, three different tumor xenograft dECM were selected to be cell-seeded. The selected samples correspond to tumor-derived dECM from the PC-3 human prostate model, PANC-1 human pancreas model, and HCC1806 human TNBC basal-like subtype model. Moreover, a commercially available collagen-based foam was included as a control (**Figure 5**).

Initial SEM imaging revealed that all dECM and control collagen foam present an open porous scaffold structure (**Figure 5A**; **Supplementary Figure S2**). Three days after cell seeding, substrate-attached live cells were detected on all samples (**Figure 5B**, green cells correspond to calcein live dye positive cells). After sample fixation, cell morphology was analyzed by detecting the area of the cells (**Figure 5C**, actin cytoskeleton staining in orange, cell nuclei in blue). Images indicate morphological differences related to seeding substrate, with more spread cell morphology and bigger cells on HCC1806 xenograft dECM substrate. Additional immunostaining studies were performed to visualize proliferative cells (**Figure 5D**, cell nuclei in blue and Ki67 proliferation marker observed as green dots at cell nuclei area). Quantitative data were obtained for cell morphology (**Figure 5E**), and cell proliferation (**Figure 5F**) and data show statistically significant differences related to cell size and proliferation rate of the cells seeded on the HCC1806 dECM substrate.

**Figure 6:**
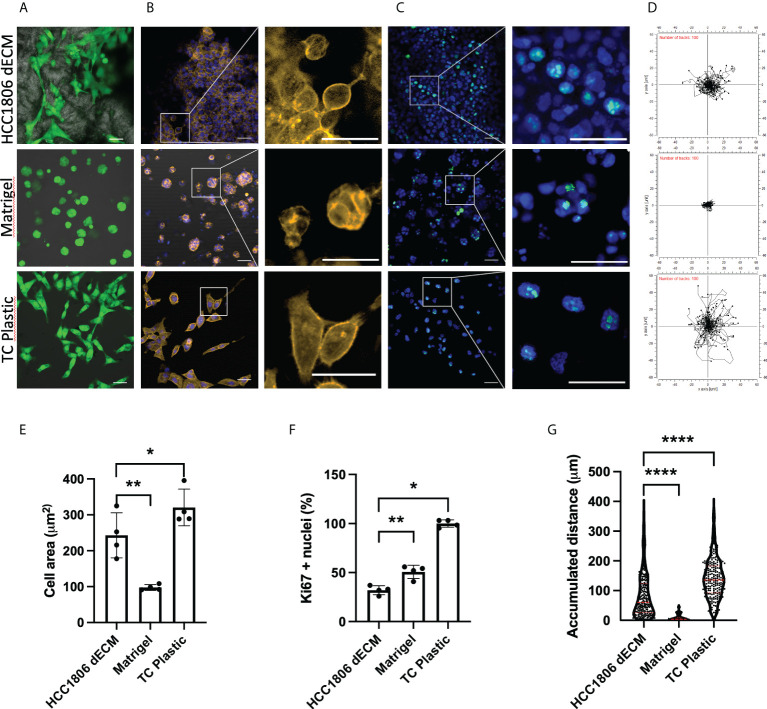
HC1806 cells show different cell behaviors on HC1806 dECM compared to other 2D and 3D culture methods. **(A–C)** Images of cell-seeded samples. **(A)** Calcein-AM–positive cells are shown in green, while scaffold material is shown in grey (×20 (scale, 20 μm)). **(B)** Actin cytoskeleton immunostaining in orange; DAPI nuclear staining in blue (×10 (scale, 20 μm) and detailed images (scale, 50 μm)). **(C)** Ki67 immunostaining in green; DAPI nuclear staining in blue (×10 (scale, 50 μm) and detailed images (scale, 20 μm)). **(D)** Plotting of the cell displacement during 5 h. Each line represents one cell; 100 cells are provided per plot. The starting point of each cell is provided centered for easy visualization. **(E)** Quantification of cell size (area) using actin cytoskeleton membrane distribution images. **(F)** Quantification of proliferative cells (Ki67-positive cells vs. total cells observed in each material). **(G)** Quantification of cell displacement measured by accumulative distance traveled. *N* = 3 in all cases and 2 areas measured per sample.

Similar cell staining and measurement tools were used to define any potential cell-behavior difference between HC1806 xenograft dECM and other common 2D and 3D substrates, such as plastic cell culture substrate or Matrigel 3D cell culture methods. Live cells were observed in all cell-seeding models (**Figure 6A**, green cells correspond to calcein live dye positive cells). After fixation, actin cytoskeleton staining (**Figure 6B**, actin cytoskeleton staining in orange and cell nuclei in blue) and Ki67 proliferative maker staining (**Figure 6C**, cell nuclei in blue and Ki67 observed as green dots at cell nuclei area) were performed. Images indicate differences in cell morphology with more pronounced morphology in 2D cultures and dECM matrix compared to spheroid formation in Matrigel cell cultures. Quantitative data for cell morphology (**Figure 6E**) and cell proliferation (**Figure 6F**) show statistically significant differences in the measured parameters. Additionally, displacement of calcein-positive live cells was recorded and plotted (**Figure 6D**; **Supplementary Figure S3**), suggesting cells at Matrigel barely move. Quantification in **Figure 6G** indicates statistically significant differences related to cell movement.

## Discussion

Cancer solid mass decellularization has been tested before in different research contexts and for multiple research purposes (35). In this manuscript, we aimed to prove that xenografted tumor-derived dECM may be achieved and used as a substrate on which the same cell line can be seeded again, thereby yielding a biomimetic, tumor-cell context-simulating, *in vitro* 3D cell culture system. For this purpose, we generated a multidisciplinary research approach compiling; animal research for xenotransplantation and generation of the raw tumors; material research for decellularization of samples; proteomics for sample characterization; and *in vitro* cell culture studies to prove the feasibility and outcome of the approach.

The previous bibliography in the tumor decellularization field tends to show studies decellularizing one specific type of tumor, with scarce research on optimization of the decellularization protocol and usually reporting sequential enzymatic- (trypsin) and detergent-based (Triton X-100) decellularization (48, 49). On the contrary, in the physiological tissue decellularization context, the development of tissue-specific decellularization protocols has gained relevance, and multiple decellularization protocol comparative research studies are common (50–52). Therefore, in our research approach, we defined the initial objective of the study and defined a decellularization protocol broadly useful in multiple solid tumor contexts.

In this sense, we generated a cohort of 13 different xenografted tumor cell lines, corresponding to seven different tissues of origin (**Table 1**). We then chose one of them to test some of the most common decellularization procedures, e.g., trypsin-EDTA and Triton X-100, in xenografted tumors (**Figure 1**). As controls, we included protocols with only the complementary reagents or treatments, such as multiple freezing of the raw tissue to easy cell detachment from the ECM; DNAse treatment to eliminate cell waste; and EDTA treatment as ion chelator to break integrin-ECM interaction. Note that EDTA is considered a gentler cell detaching agent when used in a cell culture context, and it is generally used in combination with trypsin, a protease effective for cell detachment purposes. Interestingly, the combination of multiple freezing with EDTA and DNAse treatments resulted in enough to induce decellularization without the need for trypsin, which is potentially deleterious for the ECM proteins, or Triton X-100, a detergent potentially harmful for the ECM, too. The histology study revealed not only decellularization with preservation of collagen matrix but also the preservation of noncollagenous proteins stained by Fast Green, an ECM component absent when samples were treated with trypsin or Triton X-100 (**Figure 1C**). Freezing-EDTA-DNAse treatment was further tested in all xenografted tumor types, and this approach yielded the successful decellularization of all tested samples (**Figure 2**).

In order to characterize the obtained tumor-derived dECM and define possible differences between xenografted tumors, a proteomic analysis was performed (**Figure 3**). Proteomics is a powerful tool to define the composition of a sample, in this case, dECM, but to our knowledge, it has not been applied to the study of that kind of sample before. We were able to detect thousands of individual proteins in each sample, most of them related to the extracellular matrix, extracellular vesicles, and extracellular location (**Figures 3A, B** **)**. Interestingly, proteomic data from all tumor-derived samples were notably different from Matrigel, the 3D environment used to generate the implants, indicating the ECM composition develops during tumor formation and changes compared to this tumor cell-line-derived ECM (**Figure 4A**). While DNA and nuclear proteins were removed with the decellularization, it is worth noticing that proteomic study reveals the existence of cellular organelle waste material from mitochondria and endoplasmic reticulum in tumor-derived samples (**Figure 3B**).

Proteomics data analysis also revealed a different dECM composition in two of the breast cancer samples, HCC1806 and 4T1, compared to all other dECM tumor xenograft samples (**Figure 4**). The breast cancer samples included in this study correspond to the triple-negative subtype, and among them, MDA-MB-231 cells have been previously classified as mesenchymal stem-like subtype, while HCC1806 cells represent the basal-like subtype (53). A recent study based on the multiomics characterization of the mouse 4T1 TNBC cell line found significant overlap with top pathways and GO terms reported for the basal-like TNBC subtype (54). Therefore, the proteomic data here presented revealed the tumors with basal-like TNBC subtype were similar between them and different from the other dECM obtained from other xenografted tumors. A detailed study shows some specific features related to lipidic metabolism and cholesterol metabolism (**Figure 4C**). These processes are mainly related to mitochondria and endoplasmic reticulum, suggesting organelle waste contained in the decellularized sample may have an influence on dECM tumor-specific composition. This relationship would go unnoticed without the proteomic study here performed. It is well-known that fatty acid synthesis is upregulated in TNBC, while specifically basal-like TNBC subtypes overexpress genes involved in the utilization of exogenous fatty acids (55). The dependency of this type of tumor on cholesterol has been assessed before, by proving that the downregulation of the cholesterol metabolism suppresses its growth. Moreover, the esterification of cholesterol has been associated with the metastasis of this specific breast cancer subtype (56–58). Therefore, these data indicate that, after decellularization, some tumor-specific characteristics can be retained and observed by proteomics, such as the upregulated lipidic metabolism at the basal-like TNBC subtypes.

We next wondered whether this specific composition of dECM in basal-like TNBC subtype samples, with some debris of mitochondria and endoplasmic reticulum related to specific lipidic and cholesterol metabolism, would have any influence on *in vitro* cell behavior when used as a substrate. Tumor-derived dECM has previously been used as a substrate for *in vitro* cell culture studies and cancer modeling. In some reports, it has been processed as hydrogels and further as inks for the printing and development of 3D bioengineered structures (34, 59). In other reports, tumor-derived dECM has been used as a 3D scaffold for cell-seeding purposes (48, 49, 60–63). Therefore, we considered using dECM directly as a substrate for seeding basal-like TNBC cells, aiming to test any substrate specificity. To our knowledge, this is the first time that a xenografted tumor dECM with no postprocessing treatment has been further tested *in vitro* with the same cell line, aiming to define any substrate vs. cell-specific behavior.

Aiming to generate data with more potentially translational meaning, we chose the HCC1806 human cell line as a testing model in further *in vitro* cell culture studies rather than the 4T1 mouse cell line. We used as substrate not only dECM from hCC1806 xenograft tumors but also dECM from other xenografted tumors types, such as the PC-3 human prostate model and PANC-1 human pancreas model, which showed a different proteomic composition compared to the basal-like TNBC subtype samples, but similar between them and also to MDA-MB-231 mesenchymal stem-like TNBC subtype (**Figure 3**). As a control, we included a collagen matrix, which should not provide any tumor-specific signal to the seeded cells. Interestingly, data indicate that HCC1806 cells spread and proliferate more in their own xenograft-derived dECM compared to dECM from other tumor xenografts or to the collagen scaffolds (**Figure 5**). This data suggest that cells recognize their own dECM, and behave specifically on it. As a complementary *in vitro* testing, we compared cells’ behavior at their own xenograft tumor-derived dECM, or embedded in Matrigel which provides multiple growth signaling, or in the conventional 2D plastic surface which does not provide any 3D environment. Again, cells show different size, proliferation, and mobility features in the tested cell culture models (**Figure 6**).

The most notable limitations of the study are related to the availability of the raw sample. The experimental approach is time consuming, as it requires medium- to long-term mouse xenotransplantation in an ectopic location to obtain the raw tumor material. It may raise ethical concerns related to experimentation in animals and samples may differ from human primary tumor tissue samples. Moreover, relatively small-sized xenograft tumors are obtained, limiting sample availability for further multiple studies. Decellularized tumor pieces are directly used for the intended application, and it avoids the potential loss of material related to postprocessing steps required to form hydrogels. However, samples are more prone to being contaminated, and the setting up of specific, more complex sterilization protocols is required.

Altogether, we believe this approach is a valid proof-of-concept to show the relevance of optimizing the tumor decellularization protocol to generate an optimal tumor dECM material useful to study tumor-specific cell–matrix interactions. This research opens the way to further use of well-characterized xenografted tumor dECM materials as a 3D cell culture platform for basic tumor biology and tumor progression studies, as well as global gene expression studies, drug testing studies, and other *in vitro* 3D cell culture functional studies relevant in the cancer field. Moreover, it may pave the way to further patient-specific physiologically relevant *in vitro* 3D research models, in which optimized decellularization may lead to primary tumor cell testing on their own dECM, thereby providing a potentially useful tool for personalized medicine research and diagnosis purposes (37, 49, 60–62, 64).

## Conclusions

A soft treatment using Freeze-EDTA/DNAse allows decellularization of the solid tumor mass of multiple types of xenografted cell lines. Interestingly, samples decellularized from basal-like triple-negative breast cancer subtype models show different proteomic ECM composition compared to the rest of the xenograft tumors tested. The *in vitro* recellularization of HCC1806 xenograft-derived dECM with HCC1806 cells yields different proliferation and cell spreading, compared to the cell behavior on dECM from other tumor origins, collagen matrices, Matrigel, or plastic cell culture surfaces. Altogether, data indicate that decellularized xenograft tumors are a feasible substrate for re-seeding purposes, thereby promoting specific cell behavior in the TNBC context. These data serve as a proof-of-concept for further potential generation of patient-specific *in vitro* research models.

## Data availability statement

The datasets presented in this study can be found in online repositories. The names of the repository/repositories and accession number(s) can be found below: ProteomeXchange Consortium, [PXD034597].

## Ethics statement

The animal study was reviewed and approved by Ethics Committee of CICbiomaGUNE.

## Author contributions

Conceptualization and methodology: AA Formal analysis: GI, UM, BA, and MA Investigation: GI, UM, BA, AR-D-A, and KB Validation and data curation: AA Writing and original draft preparation: UM, BA, NK, and AA. Visualization: FE, BO, VG-V, JL, and AA Writing, review, and editing: AA Supervision, funding acquisition, resources, and administration: BO, VG-V, JL, and AA. All authors have read and agreed to the published version of the manuscript.

## Funding

Grant RTI2018-101708-A-I00 funded by MCIN/AEI/10.13039/501100011033 and by ERDF A way of making Europe. Grants RYC2018-025502-I and PRE2018-084542 are funded by MCIN/AEI/10.13039/501100011033 and by ESF Investing in your future. Grant MDM-2017-0720 Maria de Maeztu Units of Excellence Program funded by the Spanish State Research Agency. Grant KK-2019/00093 Elkartek program funded by Basque Government. Grant CICBMG_PhD_03_2021 funded by CICbiomaGUNE and Polymat. Grant CICBMG_PhD_05_2019 funded by CICbiomaGUNE and Polymat. 2019 Leonardo Grant for Researchers and Cultural Creators, BBVA Foundation, grant number IN[19]_CMA_BIO_0119. The BBVA Foundation accepts no responsibility for the opinions, statements, and contents included, which are entirely the responsibility of the authors.

## Conflict of interest

The authors declare that the research was conducted in the absence of any commercial or financial relationships that could be construed as a potential conflict of interest.

## Publisher’s note

All claims expressed in this article are solely those of the authors and do not necessarily represent those of their affiliated organizations, or those of the publisher, the editors and the reviewers. Any product that may be evaluated in this article, or claim that may be made by its manufacturer, is not guaranteed or endorsed by the publisher.
