# Supplementary material for: Decellularization of xenografted tumors provides cell-specific in vitro 3D environment
Source: Front Oncol. 2022 Aug 18;12:956940. doi: 10.3389/fonc.2022.956940 (PMC9434107; doi:10.3389/fonc.2022.956940)
Supplement: Supplementary file 1 [file DataSheet_1.docx]

Supplementary Material

Decellularization of xenografted Tumors provides cell-specific in vitro 3D environment

Gaia Iazzolino ^1, ‡^, Unai Mendibil ^1,2, ‡^, Blanca Arnaiz ^1, ‡^, Ane Ruiz-de-Angulo ^1^, Mikel Azkargorta ^3^, Kepa Belloso ^1^, Neda Khatami ^1,^ Felix Elortza ^3^, Beatriz Olalde ^2^, Vanessa Gomez ^1^, Jordi Llop ^1^, Ander Abarrategi ^1,4,*^

^1^Center for Cooperative Research in Biomaterial (CIC biomaGUNE), Basque Research and Technology Alliance (BRTA), Donostia-San Sebastian, Spain

^2^TECNALIA, Basque Research and Technology Alliance (BRTA), Donostia-San Sebastian, Spain

^3^Center for Cooperative Research in Biosciences (CIC bioGUNE), Basque Research and Technology Alliance (BRTA), Derio, Spain

^4^Ikerbasque, Basque Foundation for Science, Bilbao, Spain

*** Correspondence:**Ander Abarrategi
aabarrategi@cicbiomagune.es

Keywords: Tumor Xenograft, decellularization, in vitro, 3D model, Breast cancer.

## Supplementary Figures and tables:

**Supplementary table 1. Statistical data related to data provided at figure 1b:**

|  | **Mean Diff.** | **95.00% CI of diff.** | **Significant?** | **Summary** | **Adjusted P Value** |
| --- | --- | --- | --- | --- | --- |
| **Control vs. Protocol a** | 98,61 | -63.76 to 261 | No | ns | 0,4537 |
| **Control vs. Protocol b** | 467 | 304.7 to 629.4 | Yes | **** | <0.0001 |
| **Control vs. Protocol c** | 447,7 | 307.1 to 588.3 | Yes | **** | <0.0001 |
| **Control vs. Protocol d** | 432,8 | 292.2 to 573.4 | Yes | **** | <0.0001 |
| **Control vs. Protocol e** | 506,4 | 344 to 668.8 | Yes | **** | <0.0001 |
| **Control vs. Protocol f** | 507,6 | 345.2 to 669.9 | Yes | **** | <0.0001 |

**Supplementary table 2. Statistical data related to data provided at figure 2:**

|  | **Mean Diff.** | **95.00% CI of diff.** | **Significant?** | **Summary** | **Adjusted P Value** |
| --- | --- | --- | --- | --- | --- |
| **Control 1 vs. Protoc. e 1** | -157.3 ± 5.276 | -171.9 to -142.6 | Yes | **** | <0.0001 |
| **Control 2 vs. Protoc. e 2** | -71.97 ± 1.512 | -76.17 to -67.78 | Yes | **** | <0.0001 |
| **Control 3 vs. Protoc. e 3** | -248.5 ± 2.934 | -256.7 to -240.4 | Yes | **** | <0.0001 |
| **Control 4 vs. Protoc. e 4** | -312.3 ± 31.03 | -398.5 to -226.2 | Yes | *** | 0,0005 |
| **Control 5 vs. Protoc. e 5** | -737.7 ± 31.45 | -825 to -650.4 | Yes | **** | <0.0001 |
| **Control 6 vs. Protoc. e 6** | -1834 ± 218.8 | -2441 to -1226 | Yes | ** | 0,0011 |
| **Control 7 vs. Protoc. e 7** | -407.8 ± 20.5 | -464.7 to -350.9 | Yes | **** | <0.0001 |
| **Control 8 vs. Protoc. e 8** | -1186 ± 31.99 | -1275 to -1097 | Yes | **** | <0.0001 |
| **Control 9 vs. Protoc. e 9** | -256.7 ± 18.87 | -309.1 to -204.3 | Yes | *** | 0,0002 |
| **Control 10 vs. Protoc. e 10** | -65.29 ± 1.409 | -69.2 to -61.37 | Yes | **** | <0.0001 |
| **Control 11 vs. Protoc. e 11** | -746.1 ± 83.11 | -976.9 to -515.4 | Yes | *** | 0,0009 |


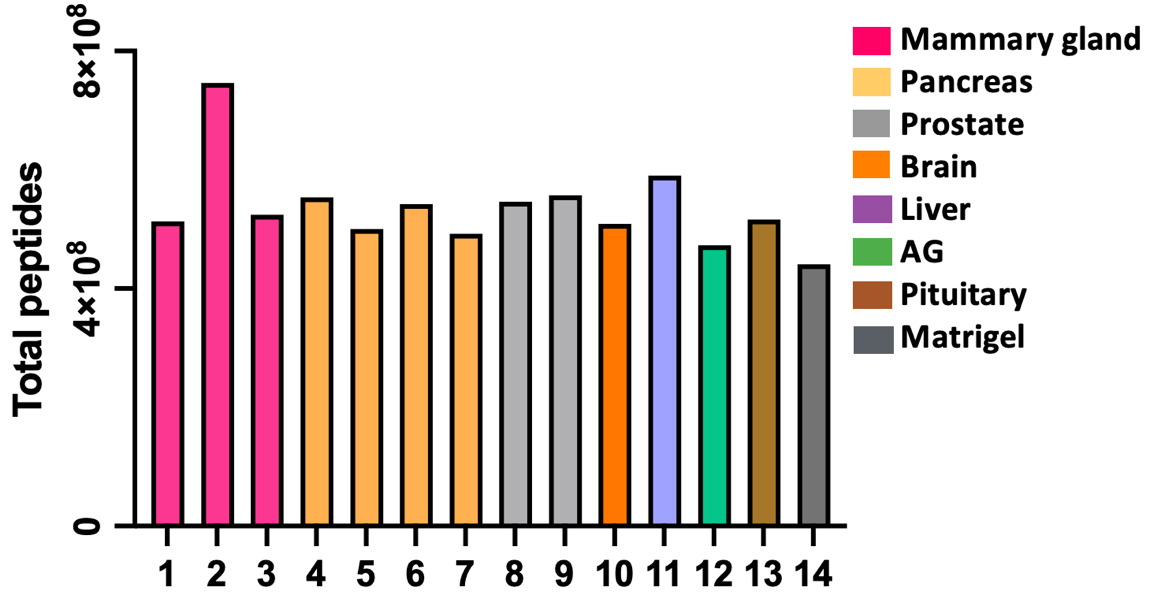


**Supplementary Figure 1.** total peptides detected per sample type in the proteomic study.


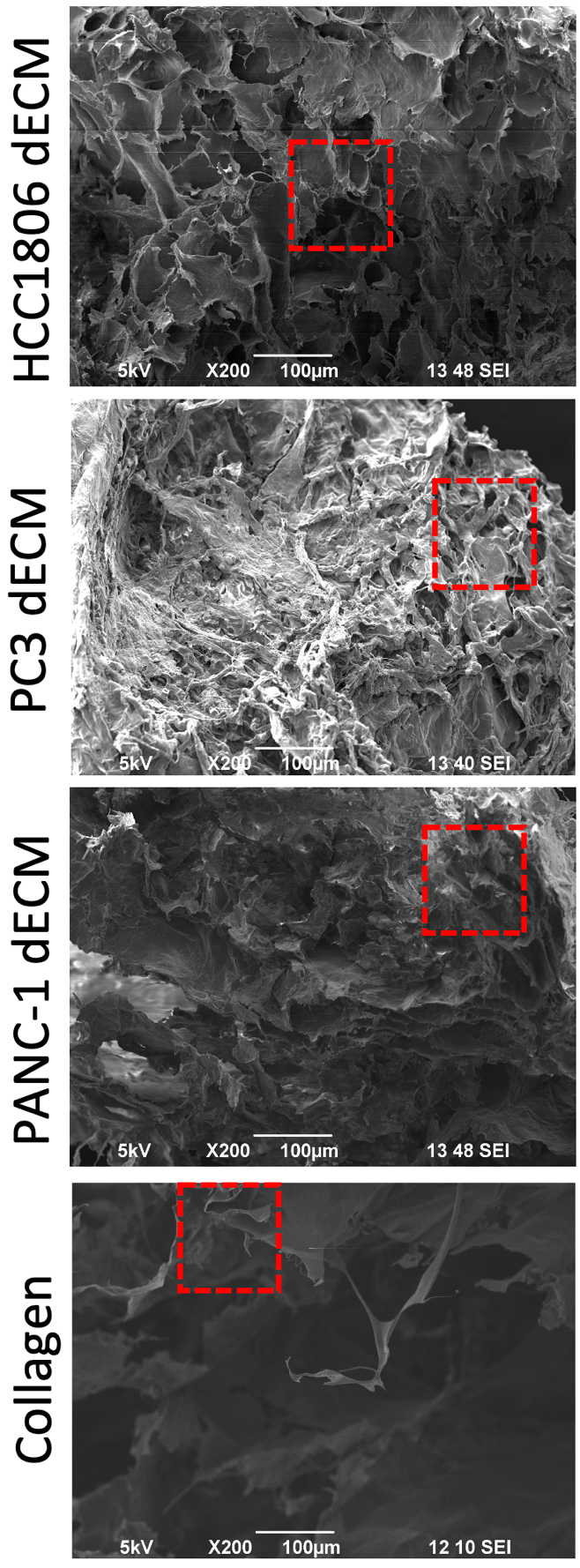


**Supplementary Figure 2. SEM images**. Red square in each image represents the area shown in figure 4a.


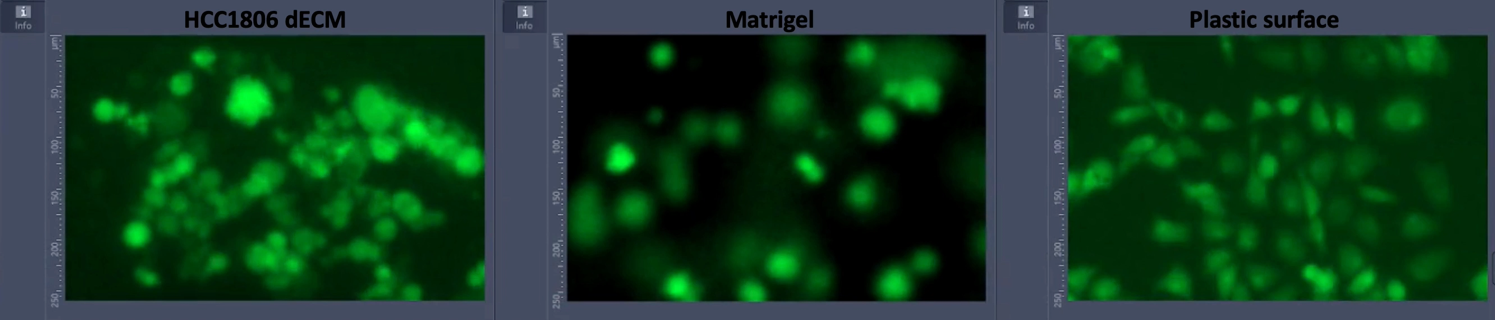


**Supplementary Figure 3. Video time-lapse imaging of HCC1806 fluorescent cells seeded in different substrates.** HCC1806 mammary tumor cells were seeded in HCC1806 tumor-xenograft dECM, Matrigel or 2D plastic cell culture surface. After 3 days, the cells were stained with Calcein-AM to visualize live cells in green, placed in an incubator chamber (Zeiss) and imaged every 15 min for 5h.
